# Immune Activation and Glycolytic Responses to *Cutibacterium acnes* Cell Wall Polysaccharides

**DOI:** 10.1016/j.jid.2025.03.045

**Published:** 2025-05-05

**Authors:** Min Qin, Evyatar Evron, Patrick Thanh Tran, Min Deng, Amanda M. Nelson, Jenny Kim, George W. Agak

**Affiliations:** 1 Division of Dermatology, Department of Medicine, David Geffen School of Medicine, University of California Los Angeles, Los Angeles, California, USA; 2 Department of Dermatology, Larkin Community Hospital, South Miami, Florida, USA; 3 Division of Dermatology, Department of Medicine, Harbor-UCLA Medical Center, West Carson, California, USA; 4 Department of Dermatology, Penn State College of Medicine, Hershey, Pennsylvania, USA

**Keywords:** Acne pathogenesis, Carbohydrate composition, *Cutibacterium acnes*, Monosaccharides, Polysaccharides

## Abstract

Carbohydrates are key components of many microbial cell walls and play a versatile role in immune recognition. In this study, we analyzed the carbohydrate cell wall composition of *Cutibacterium acnes* strains associated with healthy skin (denoted as C_H_) and acne-prone skin (denoted as C_A_) to understand their influence on host immune responses in acne. We identified glucose, mannose, and galactose as the primary monosaccharides, with minor amounts of fucose, N-acetylgalactosamine, and N-acetylglucosamine. Linkage analysis revealed structural variations between C_H_ and C_A_ strains: C_H_ strains showed a balanced and diverse polysaccharide structure, whereas C_A_ strains displayed a more rigid structure with 1→4 and branched 1→6 linkages, potentially contributing to inflammatory properties. Immunostimulatory assays revealed that *C acnes* carbohydrates induced IL-6 and IL-17 but not IL-1β, highlighting the role of carbohydrate structures in influencing cytokine responses. Treatment with sodium meta-periodate impaired this immunostimulatory activity, indicating that carbohydrate integrity is crucial for immune activation. In addition, analysis of single-cell RNA-sequencing data from acne lesions revealed elevated glycolytic activity in acne lesions in comparison with that in nonlesional skin, suggesting a Warburg-like effect that promotes inflammation. Our findings highlight the role of *C acnes* polysaccharides in immune modulation and inflammation, suggesting their potential as therapeutic targets for acne treatment.

## INTRODUCTION

Acne vulgaris is one of the most common skin diseases that affect millions of individuals, predominately in the teenage years and early 20s, but older individuals can also be affected (Bhate and Williams, 2013; Heng and Chew, 2020). The pathogenesis of acne is linked to the combination many direct and indirect factors, including hormones, sebum secretion, microbial activities, and immune responses. The major bacterium implicated in the pathophysiology of acne is *Cutibacterium acnes. C acnes*, a Gram-positive aerotolerant-anaerobic bacterium, is distinguished from other Gram-positive bacteria by its cell wall, which has a peptidoglycan structure containing a cross-linkage region of peptide chains, including L,L-diaminopimelic acid and D-alanine. These components play an immunologically active role within the cell wall structure (Kamisango et al, 1982).

Although everyone harbors *C acnes*, not all individuals develop acne or experience it with the same severity (Degitz et al, 2007; Fitz-Gibbon et al, 2013). Phylotyping and metagenomic studies of *C acnes* have demonstrated that not all *C acnes* strains are equal; certain strains are associated with acne (C_A_), whereas others are linked to healthy skin (C_H_), suggesting that strains may contribute differently to acne pathogenesis (Barnard et al, 2016; Fitz-Gibbon et al, 2013; Lomholt and Kilian, 2010; McDowell et al, 2011). These strains differ in their immunogenic potential and secretome profiles (Holland et al, 1993; Kolar et al, 2019; Suh and Kwon, 2015). Studies, including our own, have demonstrated that *C acnes* is a potent inducer of inflammatory cytokines IL-1β, IL-17, and IFN-γ from PBMCs, with C_A_ strains eliciting higher levels of these inflammatory cytokines than C_H_ strains (Agak et al, 2018, 2014; Kistowska et al, 2015; Walters et al, 1995). This suggests that different *C acnes* strains may express distinct antigenic cell wall components or ligands that modulate immune responses in the skin.

The specific *C acnes* cell wall components/ligands that mediate interactions with skin immune cells are still unclear, but the composition of its cell wall polysaccharides has emerged as a potential determinant in its ability to modulate host immune responses. Previous studies suggest that the immune-stimulatory potential of *C acnes* may be due to the presence of cell wall–derived peptidoglycan-polysaccharide polymers, which induce the secretion of proinflammatory cytokines, likely through toll-like receptor (TLR) 2 activation (Holland et al, 1993; Kim et al, 2002; Vowels et al, 1995). *C acnes* peptidoglycan components may also activate complement and the modulation of costimulatory molecules and TLR expression on antigen-presenting cells, thereby influencing T helper cell responses (Squaiella et al, 2008; Squaiella-Baptistão et al, 2015; Webster et al, 1985).

In this study, we tested the hypothesis that cell wall components from distinct *C acnes* strains contain complex carbohydrate structures that contribute to differential immune responses in acne vulgaris. Although our analyses primarily capture the overall polysaccharide composition of these strains rather than exclusively measuring isolated cell wall polysaccharides, they provide critical insights into strain-specific carbohydrate profiles. Using advanced carbohydrate analysis techniques, including high-performance anion-exchange chromatography with pulsed amperometric detection and gas chromatography-mass spectroscopy (GC-MS), we identified the monosaccharide content and linkage patterns of 6 *C acnes* strains. Our findings elucidate the polysaccharide structures in select C_H_ and C_A_ strains and explore their capacity to induce cytokine production and inflammasome activation, highlighting their distinct roles in immune activation and inflammation.

## RESULTS

### The cell wall polysaccharides of *C acnes* strains are primarily built from galactose, glucose, and mannose

**Figure 1.**
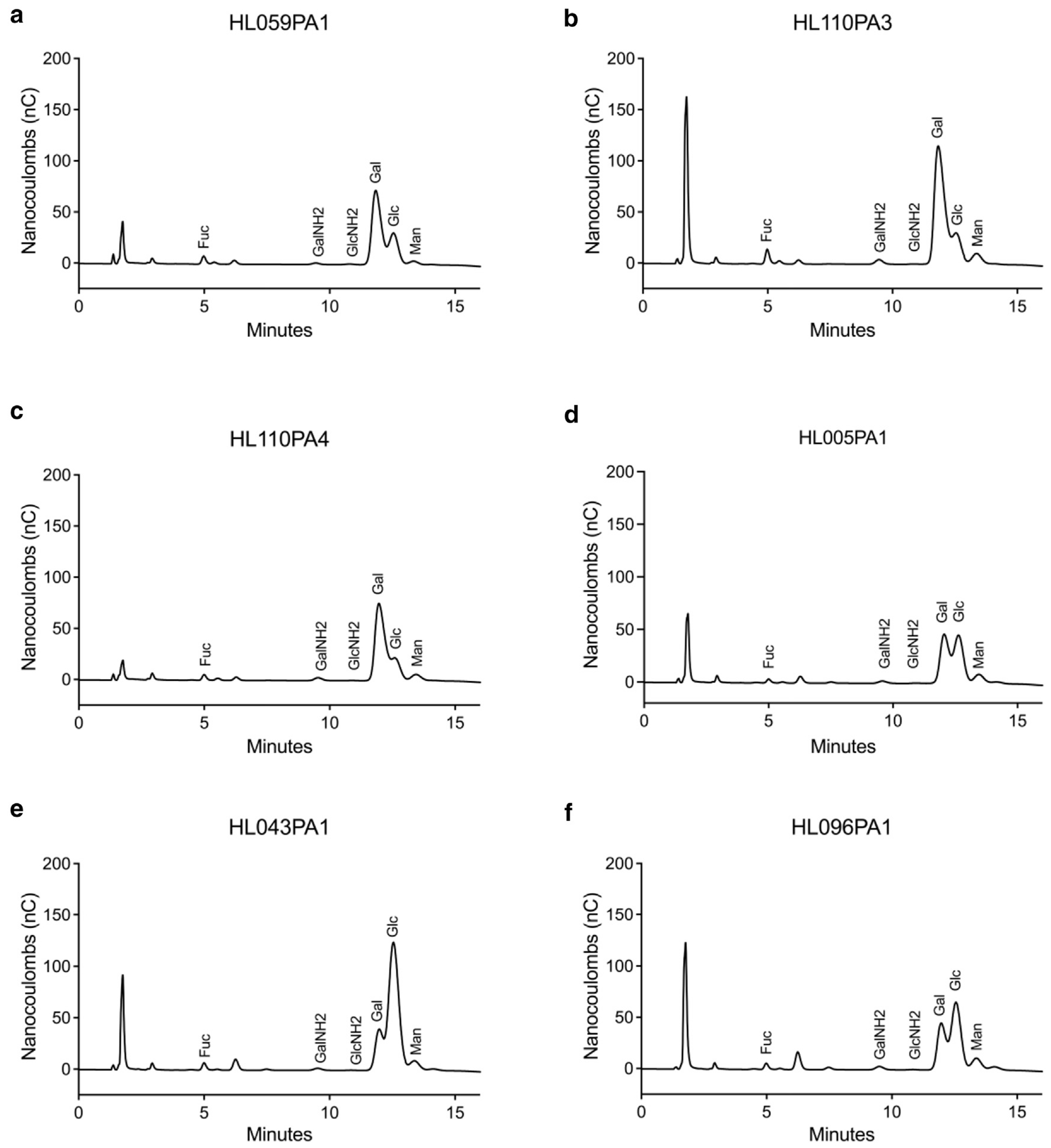
Monosaccharide composition analysis of polysaccharide isolated from healthy skin and acne-associated *C acnes* strains. (**a–c**) Polysaccharides were isolated from C_H_-associated strains (HL059PA1, HL110PA3, and HL110PA4). (**d–f**) C_A_-associated strains (HL005PA1, HL043PA1, and HL096PA1) using GC-MS. Monosaccharides present in the samples were quantified by comparing with chromatogram from known amount of standard mixture (Hardy et al, 1988; Townsend et al, 1988). Each peak in the chromatogram represents a specific PMAA, corresponding to a particular sugar, with the peak height indicating the abundance of that sugar. The retention times and peak intensities were matched to those of known standards to identify the specific sugars in the polysaccharides. Fuc denotes fucose, Gal denotes galactose, Glc denotes glucose, Man denotes mannose. GalNH2, D-galactosamine; GC-MS, gas chromatography-mass spectroscopy; GlcNH2, D-glucosamine; PMAA, per-O-methylated alditol acetate.

Early typing of *C acnes* identified 2 serotypes that categorized the bacterial species on the basis of differences in cell wall sugars (Johnson and Cummins, 1972). These serotypes, type I and type II, induced distinct antibody responses. Recent studies have suggested that type I strains are more commonly associated with acne-prone skin, whereas type II strains are more frequently found on healthy skin (Lomholt and Kilian, 2010; Spittaels et al, 2020). We therefore investigated differences in carbohydrate cell wall composition of 6 *C acnes* strains associated with healthy skin (C_H_) and acne-prone skin (C_A_) using GC-MS. We identified the monosaccharides isolated from 6 *C acnes* strains focusing on retention time and mass fragmentation patterns to confirm the identities of several sugars as previously described (Hardy, 1989; Townsend et al, 1988) (Supplementary Table S1). Carbohydrate analysis showed that the monosaccharides fucose, N-acetylgalactosamine, N-acetylglucosamine, galactose, glucose, and mannose were common in the bacterial cell walls of all 6 strains with varied composition (Figure 1). For the 3 C_H_ strains (HL059PA1, HL110PA3, HL110PA4), galactose, glucose, and mannose were the primary monosaccharides, with galactose being the most dominant (Figure 1a–c). The peak heights of these 3 sugars indicated that they form the bulk of the bacterial polysaccharide cell wall. Their longer retention times reflected their higher molecular weights and/or lower polarities. On the other hand, fucose, N-acetylgalactosamine, and N-acetylglucosamine appeared earlier in the retention timeline with lower peak heights, indicating their lower quantities, because they represented minor components in the overall polysaccharide structure. We observed similar polysaccharide composition in C_A_ strains (HL005PA1, HL043PA1, HL096PA1), with glucose and galactose being the most important monosaccharides in the structure, followed by mannose (Figure 1d–f). The minor monosaccharides (fucose, N-acetylgalactosamine, and N-acetylglucosamine) also followed the same pattern, suggesting a shared pathway or functional role in the bacterial polysaccharide architecture. Strains HL110PA3 and HL043PA1 had the highest galactose and glucose monosaccharide content on the basis of the peaks, respectively. Overall, glucose and galactose and, to a lesser extent, mannose dominated the polysaccharide composition across all strains. These 3 sugars likely make up the primary backbone of the *C acnes* polysaccharides. Slight variations in the relative amounts of each monosaccharide between strains might reflect strain-specific functions.

We next quantified the monosaccharide content of the 6 *C acnes* strains. In 4 of the strains (HL059PA1, HL110PA3, HL110PA4, and HL005PA1), galactose emerged as the primary monosaccharide displaying the highest peaks, followed by glucose and mannose. Although we did not directly assess carbohydrate acquisition preference, degradative capacity, or synthetic ability, this finding suggests that the 4 *C acnes* strains may either preferentially acquire polysaccharides that release galactose, possess a greater capacity to break down galactose, or have enhanced ability to synthesize galactose (Supplementary Figure S1a–g). Conversely, C_A_-associated strains HL043PA1 and HL096PA1 exhibited higher levels of glucose, highlighting potential differences in polysaccharide structure among these strains. In addition, fucose, galactosamine, and glucosamine were detected in moderate quantities across all strains, with slight variations, suggesting that they may play a less prominent role in the overall polysaccharide structure of *C acnes*.

### Linkage analysis of *C acnes* strains reveals structural variations and potential ecological adaptations

**Figure 2.**
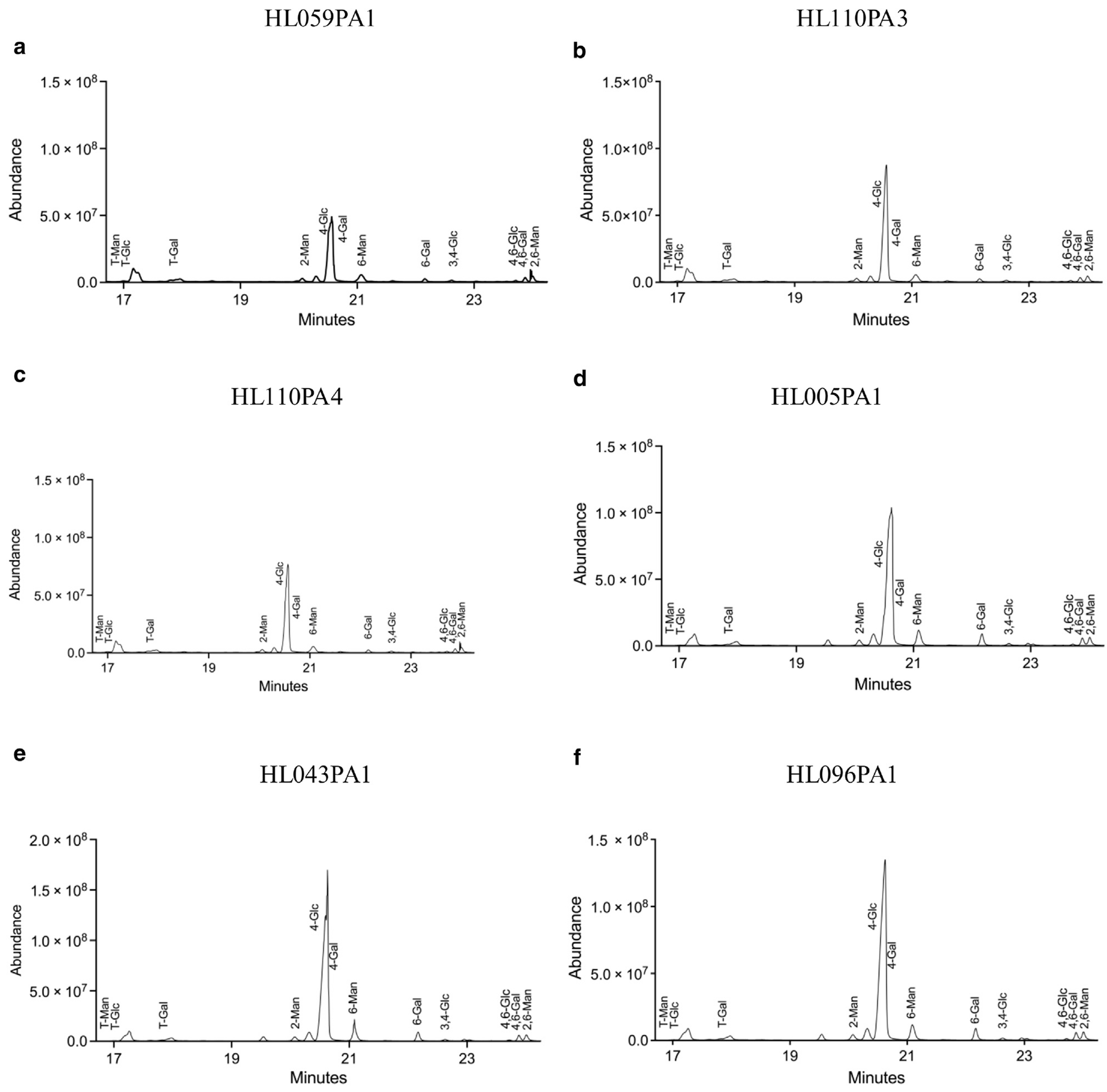
Monosaccharide linkage analysis of polysaccharide isolated from *C acnes* strains associated with healthy and acne skin. (**a–c**) Polysaccharides were isolated from C_H_-associated strains (HL059PA1, HL110PA3, and HL110PA4). (**d–f**) Polysaccharides from C_A_-associated strains (HL005PA1, HL043PA1, and HL096PA1) analyzed using GC-MS. Monosaccharides present in the samples were quantified by comparing with chromatogram from known amount of standard mixture, and the PMAAs identified through GC-MS were quantified in linkage analysis. Characterizations of the linked monosaccharides were done from the retention time and mass fragmentation patterns. In each chromatogram, specific linkages are indicated by labels next to the peaks, with each peak representing a particular glycosidic bond type. The height of the peak corresponds to the abundance of that linkage in the sample. GC-MS, gas chromatography-mass spectroscopy; PMAA, per-O-methylated alditol acetate.

We next performed GC-MS using per-O-methylated alditol acetate derivatives to analyze the linkage positions of monosaccharides in carbohydrates isolated from the 6 *C acnes* strains. The analysis revealed that C_H_ and C_A_ strains of *C acnes* exhibit distinct polysaccharide structures, with C_H_ strains showing a more balanced and complex mixture of linkages, including 1→4, 1→6, and 1→3 bonds, which contribute to structural stability and flexibility (Figure 2). In contrast, C_A_ strains predominantly featured 1→4 linkages, forming a rigid backbone with varying degrees of branching, particularly through 1→6 linkages. We also observed that the incorporation of galactose into the larger carbohydrate structures of *C acnes* cell walls differed between the C_H_ and C_A_ strains. Notably, C_A_ strains HL043PA1 and HL096PA1 demonstrated a higher prevalence of the 4,6-Gal linkage pattern, indicating a more branched polysaccharide structure (Figure 2e and f). Our in-depth analysis demonstrates that *C acnes* possesses complex glycosidic linkages within its carbohydrate cell walls, potentially explaining differences in the biological roles and/or ecological adaptations among strains. However, analysis of 4,6Glc, 4,6Gal, and 2,6Man polysaccharide levels across the 6 strains did not reveal significant peaks, suggesting that these linkages may be present at low levels or not well-resolved in the chromatographic data owing to poor signal resolution (Supplementary Figure S1h). Although 4,6-Gal appears at similar levels across all strains, the absence of distinct peak variation may be due to low intensity, overlapping peaks, or limitations in the detection processes. Overall, the linkage profiles likely reflect the functional differences between the C_H_ and C_A_ strains in their adaptation to the skin environment (Supplementary Table S2).

## *C ACNES* CARBOHYDRATES INDUCE IL-6 AND IL-17 BUT NOT IL-1β CYTOKINE SECRETION

**Figure 3.**
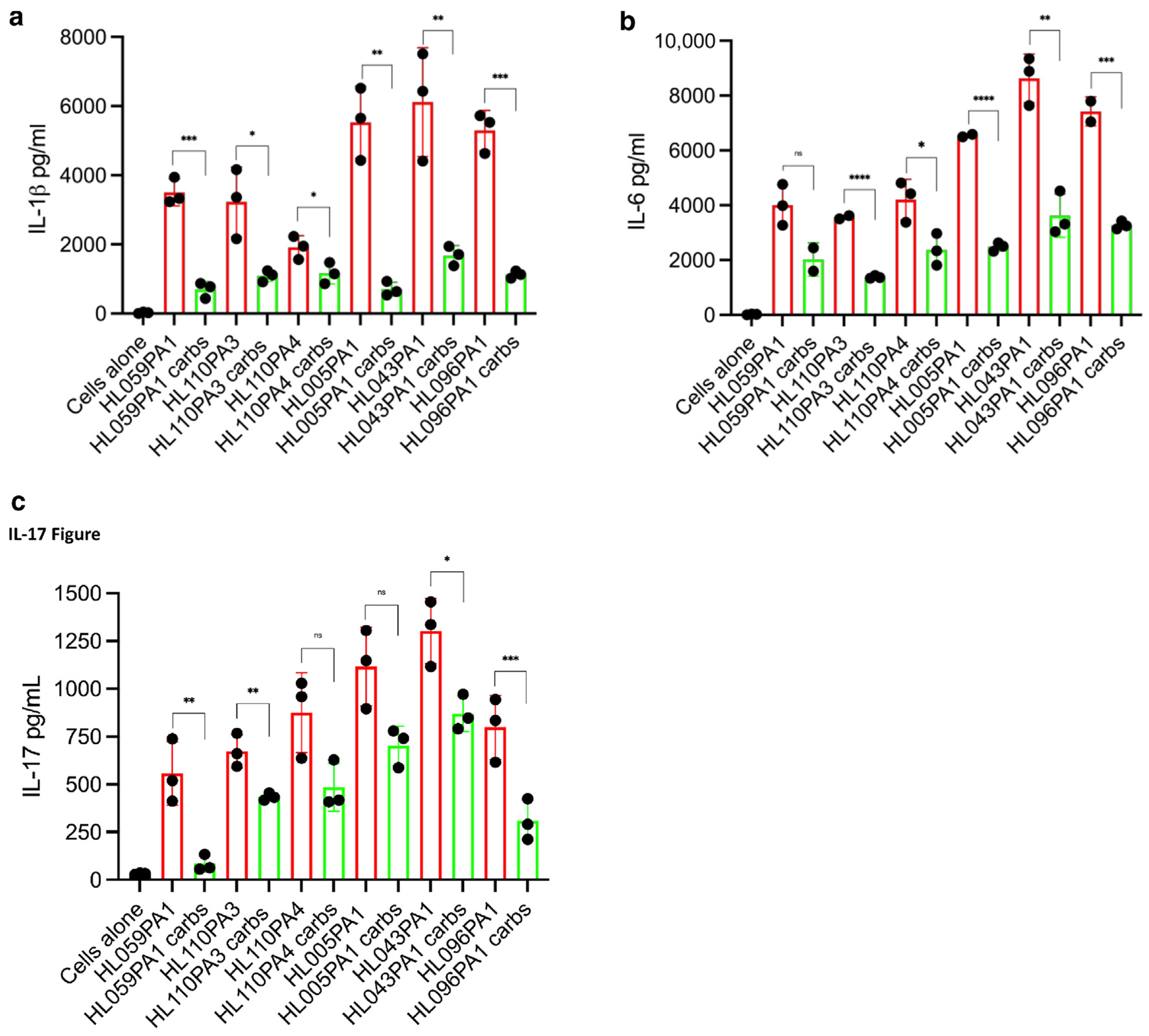
*C acnes* carbohydrates have stimulatory potential. PBMCs were stimulated with 1 μg/ml of carbohydrates isolated from *C acnes* strains associated with either healthy or acne skin. Whole bacteria from C_H_ strains (HL059PA1, HL110PA3, and HL110PA4) and C_A_ strains (HL005PA1, HL043PA1, and HL096PA1) were used at an MOI of 0.5. (**a**) IL-1β, (**b**) IL-6, and (**c**) IL-17 cytokine levels in the culture supernatants were determined by ELISA. Student’s *t*-tests were used to perform the statistical analysis. *P < .05, **P < .01, ***P < .001, and ****P < .0001. MOI, multiplication of infection; ns, nonsignificant.

The observed the differences in carbohydrate cell wall structure across the *C acnes* strains suggested that the carbohydrate components of *C acnes* may differentially modulate the host immune response. To examine the stimulatory potential of these carbohydrate components, we stimulated PBMCs from healthy donors (n = 3) as previously described (Agak et al, 2021; Yu et al, 2016). In general, we observed that whole bacteria induced higher IL-1β, IL-6, and IL-17 protein secretion by ELISA compared with isolated carbohydrates across most strains, suggesting that additional bacterial components, beyond carbohydrates, are contributing to immune activation (Figure 3). The C_A_ strains (HL005PA1, HL043PA1, HL096PA1) showed increased cytokine induction compared with the C_H_-associated strains, especially in the case of HL096PA1, which had the highest levels for all the 3 cytokines in both whole bacteria and isolated carbohydrate conditions (Figure 3 and Supplementary Figures S2 and S3). C_H_ strain HL059PA1 minimally induced IL-1β, IL-6, and IL-17 secretion in both bacteria and carbohydrate conditions, suggesting that this strain is less inflammatory, aligning with its association with healthy skin. Generally, carbohydrates isolated from both C_A_ and C_H_ strains induced lower IL-beta1 cytokine levels than IL-6 and IL-17 levels. This pattern of IL-6 and IL-17 induction was similar in all donor PBMCs that we tested.

## OXIDATION OF CARBOHYDRATES IMPAIRS THEIR IMMUNE-STIMULATORY POTENTIAL

**Figure 4.**
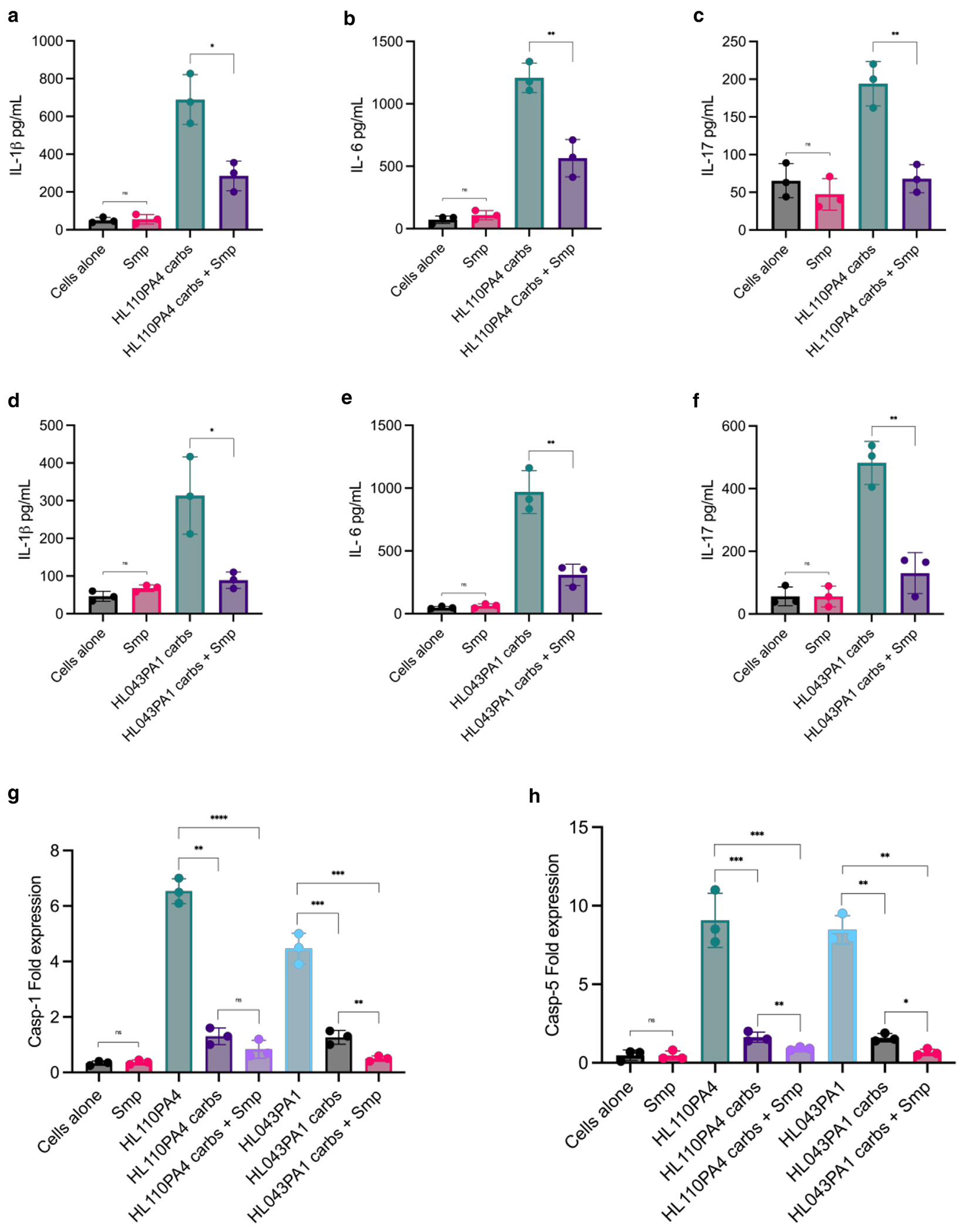
Smp treatment attenuates stimulatory potential of *C acnes* carbohydrate components and inhibits caspases 1 and 5 expressions. PBMCs were stimulated with *C acnes* at an MOI of 0.5 and 1 μg/ml of carbohydrates from (**a–c**) *C acnes* C_H_-strain HL110PA4 and (**d–f**) C_A_-strain HL043PA1 for 24 hours. IL-1β, IL-6, and IL-17 cytokine levels in the culture supernatants were determined by ELISA. (**g, h**) PBMCs were stimulated with C acnes (MOI of 0.5), carbohydrates isolated from *C acnes* strains HL110PA4 and HL043PA1, or Smp-treated carbohydrates. Real-time PCR of caspase 1 and caspase 5 mRNA expression was analyzed 24 hours after stimulation. Gene expression was normalized to *GAPDH* and quantified by the comparative method 2–ΔΔCT. Student’s *t*-tests were used to perform the statistical analysis. *P < .05, **P < .01, ***P < .001, and ****P < .0001. MOI, multiplication of infection; ns, nonsignificant; Smp, sodium meta-periodate.

We next sought to determine whether the stimulatory potential of *C acnes* carbohydrates is attenuated after treatment with sodium meta-periodate (Smp), an oxidant that converts *cis*-glycol groups in carbohydrates to reactive aldehyde groups (Webster et al, 1985). We isolated carbohydrates from *C acnes* strains HL110PA4 and HL043PA1 and used these to stimulate PBMCs from 3 healthy donors. We observed that Smp treatment significantly reduced the immune-stimulatory capacity of *C acnes* carbohydrates in both C_H_-associated strain HL110PA4 and C_A_-associated strain HL043PA1 (Figure 4). Across all measured cytokines—IL-1β, IL-6, and IL-17—the expression levels were highest when PBMCs were stimulated with whole bacteria, followed by stimulation with isolated carbohydrates, and were lowest when carbohydrates were pretreated with sodium periodate (Figure 4a–c). Smp disrupts key glycosylation patterns involved in immune activation, supporting the idea that the integrity of carbohydrate structure is necessary for optimum immune-stimulatory capacity.

### Smp-treated carbohydrates induced lower caspase 1 and caspase 5 activation

Previously, we have shown that *C acnes* induces the activation of the NLRP3 inflammasome through caspase 1 activation (Qin et al, 2014). Caspase 1, a cysteine protease, is a central player in cell immunity and in the activation of proinflammatory responses and activation of inflammatory cytokines IL-1β and IL-18. Given that the pathogenesis of acne often involves the inflammasome, we investigated whether the components of *C acnes* cell wall may trigger inflammation through inflammasome activation. To test this, we used whole bacteria, isolated carbohydrates, and Smp-treated carbohydrates from *C acnes* strain HL043PA1 to stimulate PBMCs. Whole bacteria from this strain induced significantly higher expression of caspase 1 and caspase 5 than the isolated and Smp-treated carbohydrates (Figure 4g and h). These findings strongly suggests that HL043PA1 activates both caspase 1 and 5, indicating robust activation of both canonical and noncanonical inflammasome pathways. The significantly lower expression of these caspases in response to isolated carbohydrates or disrupted carbohydrate conditions suggests that full bacterial and carbohydrate structural integrity was necessary for maximal inflammasome activation and IL-1β secretion.

### Acne lesions have enhanced glycolytic activity compared with normal skin

**Figure 5.**
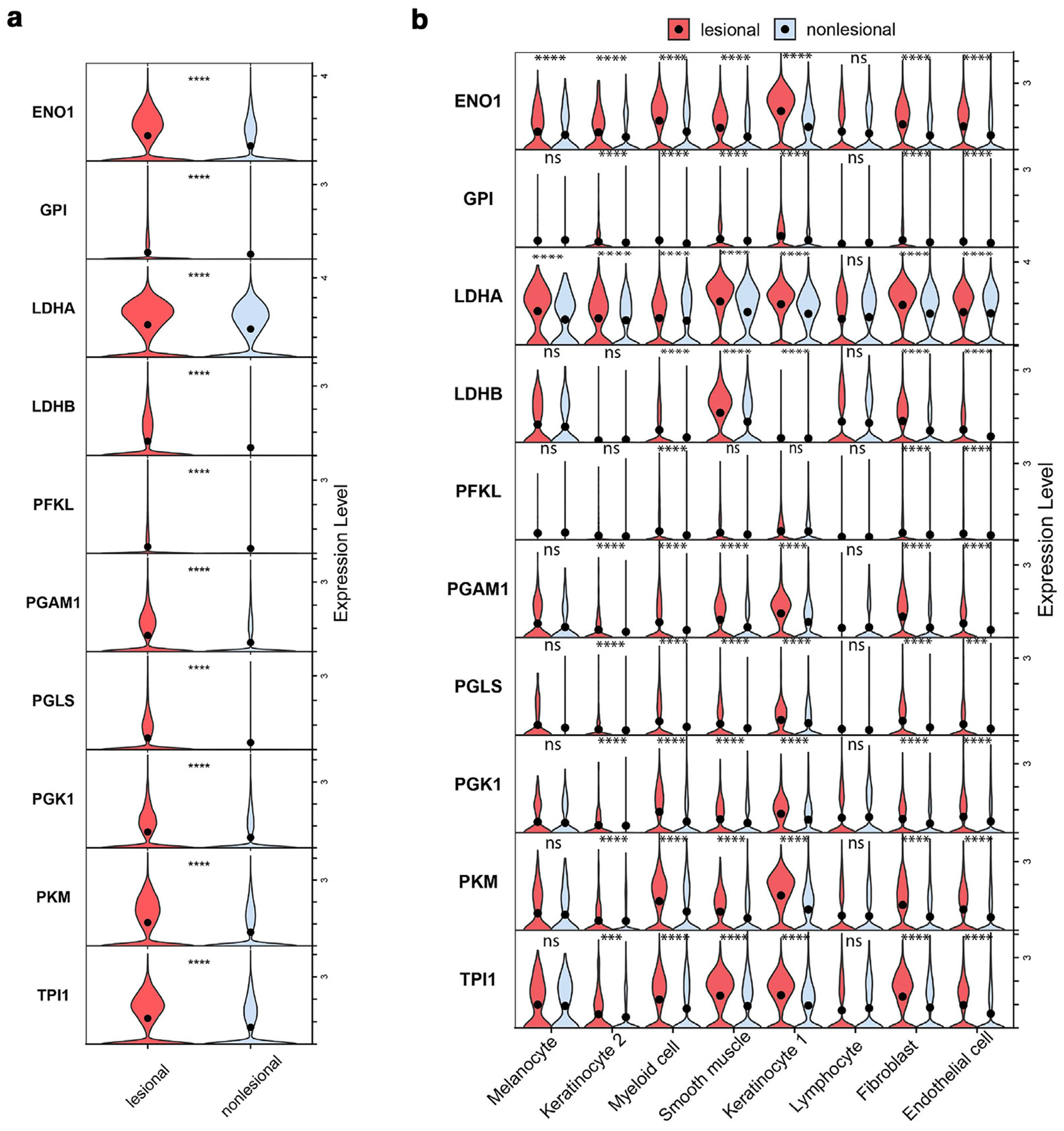
Acne lesions have enhanced glycolytic activity compared with nonlesional skin. (**a**) scRNA-seq data showing the differential gene expression of glycolytic genes in lesional compared with those in nonlesional skin. (**b**) Gene expression patterns of glycolytic genes across cell types: melanocytes, keratinocyte 1 (epidermal keratinocytes), keratinocyte 2 (sweat gland cells), myeloid cells, smooth muscle cells, lymphocytes, fibroblasts, and endothelial cells. Each cell type displays violin plots in lesional compared with those in nonlesional samples. Wilcoxon tests was used to perform the statistical analysis. ***P < .001 and ****P < .0001. ns, nonsignificant; scRNA-seq, single-cell RNA sequencing.

Carbohydrates serve as key substrates for glycolytic pathways, driving cellular energy production and influencing metabolic processes that are closely linked to immune responses and inflammation. To investigate whether acne lesions contain gene signatures that may reflect response to *C acnes* cell-wall components or polysaccharide metabolism, we analyzed our previously published single-cell RNA-sequencing (scRNA-seq) dataset, which included samples of both clinically appearing normal skin (nonlesional) and acne lesions from the backs of 6 individuals. The acne lesions were early-stage acne lesions, approximately 24 hours after onset (Do et al, 2022). The scRNA-seq dataset contains 29,202 cells spanning 8 different types: endothelial cells, fibroblasts, lymphoid cells, smooth muscle, myeloid cells, 2 keratinocyte populations (keratinocyte 1 and keratinocyte 2), and melanocytes. Our recent findings suggest active intercellular signaling among these cell types (Deng et al, 2024). We observed a significant metabolic shift in lesional skin compared with that in nonlesional skin, with upregulation of several genes involved in glycolysis, suggesting a Warburg-like effect in acne (Figure 5a) (Oliveira et al, 2015; Warburg, 1956). The increase in glycolytic activity was observed across multiple skin cell types, pointing toward an altered metabolic state that may support the increased energy demand of inflammatory cells in the acne lesions (Figure 5b and Supplementary Figure S4).

To validate our scRNA-seq findings, we confirmed *SLC2A1* (*GLUT1*) gene expression in immune cells, including TREM2 macrophages, *C acnes*-specific T helper 17 cell clones, and HaCaT keratinocytes stimulated with *C acnes* (Supplementary Figure S5). Fluorescence imaging of HaCaT cells revealed SLC2A1 (GLUT1) protein localization on the plasma membrane, indicating its potential role in glucose uptake.

Although *C acnes* stimulation did not significantly alter the percentage of SLC2A1-positive cells within the E-cadherine–positive population, the presence of *SLC2A1* in immune cells and keratinocytes suggests an active role in cellular glucose metabolism. Given that glycolytic reprogramming is a key feature of immune activation, these findings support the idea that *SLC2A1* may enhance glucose uptake in immune cells, facilitating glycolytic processes essential for immune activation during acne inflammation. Overall, the elevated expression of glycolytic genes reinforces the link between carbohydrate metabolism and immune activity, which is typically observed in acne lesions (Kelhälä et al, 2014; Kim et al, 2002; Kistowska et al, 2015).

## DISCUSSION

In this study, we investigated the carbohydrate cell-wall composition of 6 *C acnes* strains associated with either healthy or acne-prone skin and identified the primary monosaccharides that form the backbone of their polysaccharides. Our comprehensive analysis revealed that these strains have intricate glycosidic linkages within their carbohydrate cell walls and that oxidation of these carbohydrates significantly impaired their immunostimulatory activity, supporting the importance of carbohydrate structural integrity for optimal immune response. In addition, scRNA-seq data analysis from acne lesions demonstrated increased glycolytic activity and elevated expression of glycolytic genes in lesional skin compared with that in nonlesional skin. Our findings indicate that *C acnes* polysaccharides may play a role in driving strain-specific immune activation and metabolic shifts, thereby linking carbohydrate structure to acne pathogenesis.

Our analysis of *C acnes* polysaccharides revealed that glucose, mannose, and galactose were the primary monosaccharides in the cell wall, with glucose being the most abundant in both C_H_ and C_A_ strains, corroborating results from a recent study (Hao et al, 2024). Fucose, N-acetylgalactosamine, and N-acetylglucosamine were present in smaller quantities across all strains. The longer retention times of dominant monosaccharides suggest that they constitute the core structure of the bacterial polysaccharides. Notably, HL110PA3 (type II) showed elevated galactose levels, whereas HL043PA1 (type IA) had increased glucose, indicating strain-specific functions that may influence interactions with the skin environment, contributing to either skin health or acne pathology.

Polysaccharides are inherently heterogeneous, and despite advancement in analytical techniques, achieving full structural characterization remains a significant challenge (Mohammed et al, 2021). Our linkage analysis revealed significant structural differences between the polysaccharides of C_H_ and C_A_ strains, which may have important implications for their roles in skin health and disease. The balanced and diverse linkage types observed in C_H_ strains likely support skin homeostasis by promoting a commensal relationship through structural adaptability and moderated host interactions. In contrast, the C_A_ strains (HL043PA1 and HL096PA1) demonstrated a more rigid polysaccharide structure dominated by 1→4 linkages with additional branching 1→6 linkages. These structures may facilitate biofilm formation, adherence, and immune evasion within the inflamed microenvironment of acne lesions (de la Fuente-Núñez et al, 2013; Hao et al, 2024). The observed differences in linkage patterns suggest that C_A_ strains may have evolved distinct polysaccharide structures that enhance their virulence, making them more immunostimulatory than strains with simpler linkages (Barnard et al, 2016). However, our GC-MS analysis of 4,6Glc, 4,6Gal, and 2,6Man polysaccharide levels did not reveal significant peak differences, hindering our ability to tease apart the variations between strains. As a result, additional studies using complementary approaches, such as mass spectrometry with isotopic labeling or targeted glycan-specific methods, are needed to further clarify the polysaccharide linkage composition and provide a more comprehensive understanding of strain-specific adaptations to the skin environment.

In biofilms, cells are typically embedded in a matrix made up of polysaccharides, extracellular DNA, and proteins (Flemming and Wingender, 2010). These polysaccharides are crucial for mediating cell-to-surface interactions, which are key for the structural integrity and architecture of biofilms. Beyond providing structural stability, the polysaccharide matrix can protect the bacterial cells from various agents, including antibiotics (Singh et al, 2010). Interestingly, the exogenous addition of these polysaccharides was found to limit biofilm formation by Gram-positive bacteria, representing a potential strategy for biofilm prevention (Rendueles et al, 2011). Further studies are needed to identify whether targeting strain-specific polysaccharides could present novel approaches for preventing biofilm-associated persistence in acne.

The mechanisms through which carbohydrates and pathogen recognition receptor modulate immune responses are not fully understood. The immunostimulatory activity of polysaccharides may stem from direct or indirect interactions with the immune system, triggering diverse cellular and molecular events (Ferreira et al, 2015). Our study found that carbohydrates from both C_A_- and C_H_-associated *C acnes* strains induce proinflammatory cytokine activation, potentially mediated through TLR2 (Kim et al, 2002). Notably, the C_A_-strain HL043PA1 induced upregulated expression of caspase 1 and caspase 5, indicating activation of both canonical and noncanonical inflammasome pathways. The strong induction of these caspases in response to whole bacteria suggests that intact bacterial structures are necessary for maximal inflammasome activation. Previously, we demonstrated that *C acnes* triggers IL-1β secretion through the NLRP3 inflammasome activation (Qin et al, 2014). Several potential mechanisms could explain this process. One possibility is that bacterial virulence factors, such as secreted toxins or peptidoglycan fragments, gain access to the host cytosol and directly engage inflammasome sensors. Alternatively, *C acnes* may possess intracellular invasion capabilities, allowing bacterial components to enter the cytoplasm and activate inflammasomes. In addition, intracellular persistence of *C acnes* in macrophages and keratinocytes may contribute to its prolonged inflammatory effects (Fischer et al, 2013; Mak et al, 2012). Further studies examining whether *C acnes* actively invade host cells or whether bacterial-derived components and metabolites are released and recognized intracellularly will be crucial to elucidating the exact pathways involved.

Of note, carbohydrates work synergistically with other pathogen recognition receptor agonists. A study by Wilson et al (2019) reported that a synthetic random copolymer of mannose and a TLR agonist enhanced interactions between mannose and TLR7. Moreover, polysaccharides can facilitate cooperation between different pathogen recognition receptors; for instance, a glucomannan polysaccharide was found to activate both the TLR4–MD-2 complex and the mannose receptor (Li et al, 2023). As previously suggested, structural features such as polysaccharide type, molecular weight, conformation, and functional groups (eg, acetyl and sulphate groups), along with branching patterns, may contribute to immunostimulatory activity (Ferreira et al, 2015; Mueller et al, 2000). Our findings further suggest that targeting glycans capable of engaging multiple pathogen recognition receptors could be a promising strategy for modulating synergistic immune activities in the skin.

The upregulation of glycolysis-related genes (*ENO1, GPI, LDHA/B, PFKL, PGM1, PGLS, PKM, TPI1*) across multiple cell types in early-stage acne lesions suggests a metabolic shift toward aerobic glycolysis, similar to the Warburg effect observed in immune activation. This metabolic reprogramming is well-documented in immune cells, where increased glycolytic activity supports rapid energy production and biosynthetic demands essential for inflammatory responses (Escoll and Buchrieser, 2018). Although it remains unclear whether this increase in glycolytic activity in acne is directly induced by specific *C acnes* strains or driven by broader inflammatory processes, the observed metabolic shift aligns with findings in other immune-activated states, where glycolysis facilitates cytokine production and immune cell proliferation (Escoll and Buchrieser, 2018). These insights highlight potential therapeutic strategies aimed at modulating host metabolism to mitigate acne-associated inflammation and lesion development.

Our study has several limitations. *C acnes* cultures were grown under static conditions, which may have promoted biofilm formation despite multiple washing steps, adding to the complexity of the extracted polysaccharides. In addition, our polysaccharide analysis likely captured a mixture of intracellular, cell-wall–associated, and biofilm-derived polysaccharides. A previous study demonstrated that the dominant polysaccharide in the biofilm matrix of *C acnes* ribotype 5 was identical to that of its cell wall, and the authors employed rigorous methods to physically separate these components (Gannesen et al, 2019). Despite our efforts, incomplete separation of these fractions may have influenced the observed polysaccharide composition. Furthermore, although our scRNA-seq data indicate upregulation of glycolytic genes, gene expression alone does not fully capture glycolytic activity. Factors such as protein expression, post-translational modifications, substrate availability, and rate-limiting enzymatic steps also play critical roles in regulating glycolysis. Future studies incorporating functional metabolic assays, such as lactate production, extracellular acidification rate, or stable isotope tracing, could provide a more comprehensive assessment of glycolytic activity in acne-prone skin. In addition, ex vivo skin explants will be necessary to better replicate the in vivo environment and elucidate the role of *C acnes* carbohydrates in immune activation.

In summary, our findings highlight the immune-stimulatory potential of *C acnes* cell-wall carbohydrates, revealing that structural differences in polysaccharides may contribute to strain-specific immune activation in acne. The observed variations in carbohydrate linkages between C_H_ and C_A_ strains highlight the potential of polysaccharides as therapeutic targets for acne treatment. Further research is needed to determine whether targeting polysaccharide-driven immune responses could offer new strategies for mitigating acne-related inflammation and biofilm persistence.

## MATERIALS AND METHODS

### C acnes strains

*C acnes* strains (Supplementary Table S1) associated with acne skin (HL005PA1, HL043PA1, and HL096PA1) and strains associated with healthy skin (HL059PA1, HL110PA3, and HL110PA4) were obtained from BEI Resources Repository and grown in static cultures as previously described (Agak et al, 2018; Yu et al, 2016). Overnight, bacterial cultures were subcultured and incubated until mid-log phase was reached, which was determined to be optical density_600_ = 0.4. To minimize biofilm-associated carbohydrate contamination of our static cultures, bacterial cells were thoroughly washed in sterile PBS and renormalized to optical density_600_ = 0.4 in culture media (Agak et al, 2021, 2018; Yu et al, 2016). The level of endotoxin contaminating the *C acnes* was quantified with a *Limulus* Amoebocyte Lysate assay (BioWhittaker) and found to be <0.1 ng/ml^−1^.

### *C acnes*–soluble polysaccharide extraction and Smp treatment

*C acnes*–soluble polysaccharide was obtained as previously described (Squaiella et al, 2008). *C acnes* strains were cultured anaerobically for 7–14 days in Reinforced Clostridium Media until optical density readings of at least 0.7 were achieved. The strains were washed 3 times by centrifugation: each time, the bacterial cultures were centrifuged for 15 minutes at 8000 r.p.m. and then resuspended in 5 ml of Dulbecco’s PBS. The bacterial pellets were resuspended in PBS and autoclaved for 20 minutes at 120 °C. The protein concentration was determined by Bradford method. After protein concentration was determined, 10 ml of bacteria at a concentration of 1 mg/ml was mixed with 10 ml of 90% phenol and 10 ml of PBS. These phenol-treated cultures were then incubated at 70 °C for 10 minutes. After centrifugation at 4000*g* for 10 minutes under refrigeration, the supernatants were collected. The incubation was repeated with phenol and water and spun under refrigeration, and the supernatant was collected 2 more times, and then for every 1 volume of supernatant collected, 3 volumes of 100% ethanol were added. The precipitate was left to form overnight at 4 °C, which contained the soluble polysaccharide. This solution was then centrifuged at 5000 r.p.m. for 10 minutes. The supernatant was aspirated, and the pellet was resuspended with 5 ml PBS. The concentration of the total carbohydrates obtained was measured using the Abcam’s total carbohydrate quantification kit, according to the manufacturer’s instructions. The samples were then measured in a 96-well flat bottom plate at optical density of 490 nm. For Smp experiments, carbohydrates were treated with Smp (S398-50) at 10 mM for 30 minutes at 4 °C in the dark prior to PBMC stimulation.

### PBMC isolation, stimulation, and cytokine ELISAs

PBMCs were obtained from healthy donors after signed written informed consent as approved by the Institutional Review Board at University of California, Los Angeles in accordance with the Helsinki Guidelines. PBMCs were then isolated using Ficoll-Paque gradients (GE Healthcare) and plated onto 24-well tissue culture plates (2 × 10^6^/well) in RPMI 1640 media containing 10% AB serum (Gemini). Cells were cultured with different strains of live *C acnes* with multiplicity of infection of 0.5. IL-6, IL-1β, and IL-17 levels in culture supernatants were measured by ELISA, following the manufacturer’s instructions (R&D ELISA Development System ELISA kits DY201, DY206, and DY317 were used to measure IL-1β, IL-6, and IL-17, respectively). Samples were assayed in technical triplicates for each biological (donor) replicate. TREM2 macrophages and T helper 17 clones were generated as previously described (Agak et al, 2018; Deng et al, 2024).

### Monosaccharide composition analysis of the polysaccharide isolated from healthy skin and acne-associated *C acnes* strains

A total of 5 μg/ml of polysaccharide samples were hydrolyzed using 2 N trifluoroacetic acid at 100 °C for 4 hours. Excess acid was removed by dry nitrogen flush, and monosaccharides were dissolved in Milli-Q water and analyzed by high-performance anion-exchange chromatography with pulsed amperometric detection (Dionex ICS3000). Monosaccharide profiling was done using Thermo-Dionex CarboPac PA1 column (Thermo Fisher Scientific, 4 mm × 250 mm) using 100 mM sodium hydroxide and 250 mM sodium acetate as running buffer. Monosaccharides present in the samples were quantified by comparing with chromatogram from known amount of standard mixture.

### Linkage analysis of polysaccharide isolated from healthy skin and acne-associated *C acnes* strains and GC-MS

Linkage analysis and GC-MS was done at the Glycotechnology Core Resource at the University of California, San Diego (San Diego, CA). To ensure rigor and reproducibility, each GC-MS run included 3 technical replicates per sample, and biological replicates were obtained from 3 independent cultures of each strain. Briefly, 5 μg/ml amount of polysaccharide was isolated from *C acnes* strains and dissolved in anhydrous DMSO, followed by addition of sodium hydroxide slurry in DMSO and methyl iodide. Per-O-methylated polysaccharide was then subjected to hydrolysis using 4 N trifluoroacetic acid at 100 °C for 4 hours. After removal of acid, the samples were reduced using sodium borodeuteride and acetylated with mixture of pyridine and acetic anhydride. Finally, analysis of per-O-methylated alditol acetate were done using GC-MS equipped with Restek 5MS capillary column (30 m × 0.25 mm). Characterizations of the linked monosaccharides were done from the retention time and mass fragmentation patterns, allowing for precise identification.

### RNA isolation, cDNA synthesis, and real-time PCR

Total RNA was isolated from PBMCs 24 hours after stimulation using Trizol reagent (Invitrogen), following the manufacturer’s instructions, and treated with RNase-free DNase. RNA samples were reverse transcribed to cDNA using iScript cDNA synthesis kit (Bio-Rad Laboratories). Reactions were done at 25 °C for 5 minutes, 42 °C for 30 minutes, and 85 °C for 5 minutes. Real-time PCR was applied using iQ SYBR Green Supermix (Bio-Rad Laboratories). A total of 40 cycles were carried out at 95 °C for 5 minutes, then 95 °C for 10 seconds, 55 °C for 20 seconds, and 72 °C for 20 seconds. *GAPDH* was used as a control. Gene expression level was quantified by the comparative method 2^−ΔΔCT^. The list of primers used in the study is presented in Supplementary Table S3.

### scRNA-seq

scRNA-seq, alignment, and data processing were done as previously described (Deng et al, 2024; Do et al, 2022). Data visualization was conducted using Seurat (version 5.1.0). To identify carbohydrate/glycolysis-associated genes that were differentially expressed between nonlesional and paired lesional samples from 6 donors, we applied the stat_compare_means function from the ggpubr package (version 0.6.0) with the Wilcoxon rank-sum test to determine *P*-values and significance levels. Plots were generated using ggplot2 (version 3.5.1).

### HaCaT cell culture and *C acnes* treatment

HaCaT keratinocytes were cultured in DMEM + GlutaMAX-I (Gibco, catalog number 10566-016) supplemented with 10% fetal bovine serum and penicillin/streptomycin. Cells (9 × 10^4^) were seeded onto poly-L-lysine–coated coverslips placed at the bottom of a 6-well plate and incubated at 37 °C with 5% carbon dioxide for 4 hours to allow attachment. Upon attachment, cells were stimulated with healthy (110PA3) and/or acne- (43PA1) associated strains at multiplicity of infection of 40. Cells were harvested after 4 hours and used for further experiments.

### Immunofluorescence staining of HaCaT cells

After *C acnes* treatment, the culture medium was aspirated, and HaCaT cells were washed 3 times with 1X PBS. Fixation was performed using 4% paraformaldehyde for 15 minutes at room temperature in the dark, followed by 3 additional washes with 1X PBS (5 minutes each). Cells were then blocked in a blocking buffer containing 5% normal goat serum and 0.5% Triton X-100 in PBS for 60 minutes at room temperature. Next, cells were incubated overnight at 4 °C in the dark with Alexa Fluor 488–conjugated anti–E-cadherin antibody (Cell Signaling Technology, catalog number 3199, 1:200) and Alexa Fluor 594–conjugated GLUT1 antibody (Abcam, catalog number ab20636, 1:100). Then, slides were washed twice with 1X PBS and mounted using ProLong Gold Antifade Reagent with DAPI. Coverslips were sealed with clear nail polish, and immunofluorescence imaging was performed using Leica TIRF & D-STORM/THUNDER microscope at ×20 magnification.

### Statistical analysis

Data obtained from at least 3 independent experiments were analyzed using GraphPad Prism software, version 8. Nonparametric test was used to determine significance of datasets not normally distributed. If more than 2 datasets were compared, Kruskal–Wallis H-test was used to compare variances within groups. For comparisons among 3 or more groups, we used Kruskal–Wallis H-test. Posthoc pairwise comparisons to determine group differences were performed using the Dunn’s test with Bonferroni correction. Significant differences were considered for those probabilities < 5% (*P* < .05). ns denotes not significant. *P < .05, **P < .01, ***P < .001, and ****P < .001. Kruskal–Wallis and other inferential analyses were performed using Python, version 3.13. Statistical details for each experiment are indicated in the figure legends.

## Supplementary Material

1

Supplementary material is linked to the online version of the paper at www.jidonline.org, and at https://doi.org/10.1016/j.jid.2025.03.045.

## Data Availability

The data that support the findings are available from the corresponding author upon request.

## Abbreviations:

GC-MS: gas chromatography-mass spectroscopy
scRNA-seq: single-cell RNA-sequencing
Smp: sodium meta-periodate
TLR: toll-like receptor
