# Supplementary material for: Immune Activation and Glycolytic Responses to Cutibacterium acnes Cell Wall Polysaccharides
Source: J Invest Dermatol. Author manuscript; Available in PMC 2026 Feb 6. (PMC12879912; doi:10.1016/j.jid.2025.03.045)
Supplement: 1 [file NIHMS2136639-supplement-1.pdf]

SUPPLEMENTARY REFERENCES

Agak GW, Kao S, Ouyang K, Qin M, Moon D, Butt A, et al. Phenotype and antimicrobial activity of Th17 cells induced by Propionibacterium acnes Strains associated with healthy and acne skin. J Invest Dermatol 2018;138:316–24.

de la Fuente-Núñez C, Reffuveille F, Fernández L, Hancock RE. Bacterial biofilm development as a multicellular adaptation: antibiotic resistance and new therapeutic strategies. Curr Opin Microbiol 2013;16:580–9.

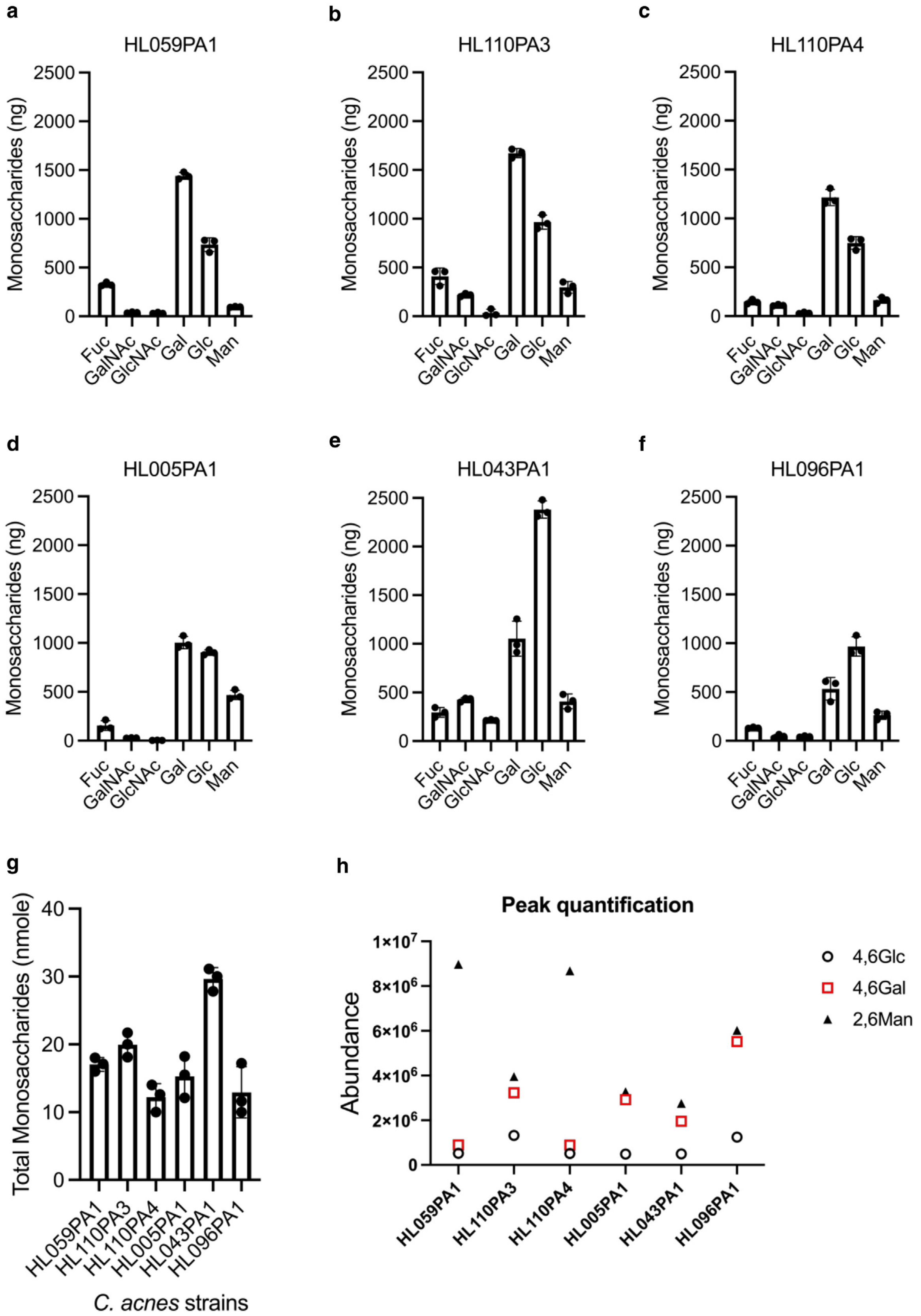

Supplementary Figure S1. Analysis of monosaccharides isolated from *C acnes* strains. (**a**–**c**) Polysaccharide samples isolated by phenol extraction from C_H strains HL059PA1, HL110PA3, and HL110PA4. (**d**–**f**) Polysaccharide samples isolated by phenol extraction from C_A strains HL005PA1, HL043PA1, and HL096PA1. Total monosaccharides in each sample were analyzed by HPAEC-PAD. (**g**) Total monosaccharides in each *C acnes* strains in nanomoles (nmole). (**h**) Peak quantification of 4,6Glc, 4,6Gal, and 2,6Man polysaccharides in 6 *C acnes* strains. Peaks corresponding to specific glycosidic linkages were identified on the basis of retention times and mass fragmentation patterns, with their relative abundances quantified. Data points represent mean peak intensities. HPAEC-PAD, high-performance anion-exchange chromatography with pulsed amperometric detection.

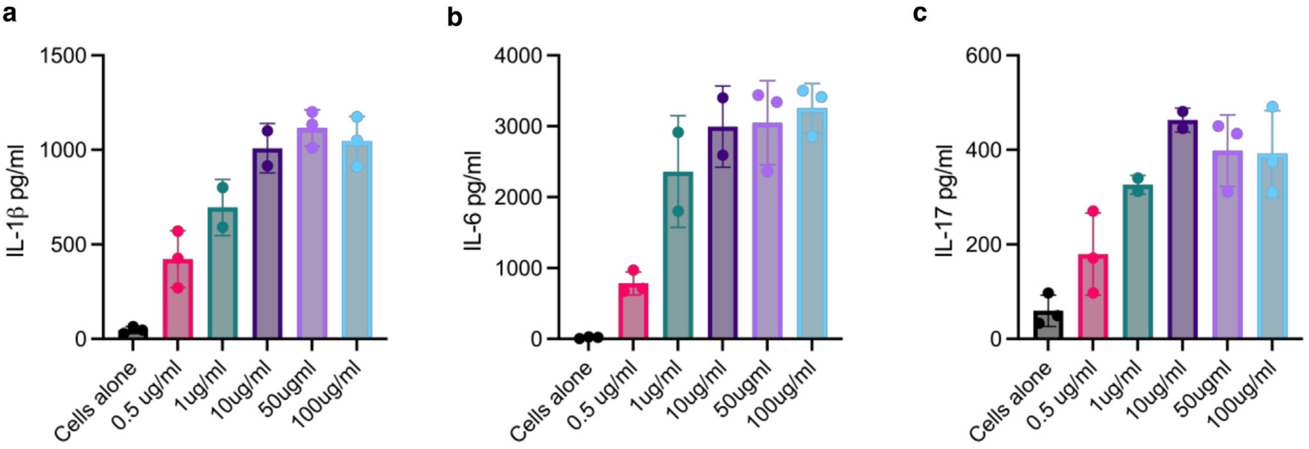

Supplementary Figure S2. Dose−response relationship of *C acnes* carbohydrates. (**a**−**c**) PBMCs (2−5 × 10^6/ml) were stimulated with various concentrations of *C acnes* carbohydrates for 24 hours, and the levels of IL-1β, IL-6, and IL-17 secretion was determined using ELISA.

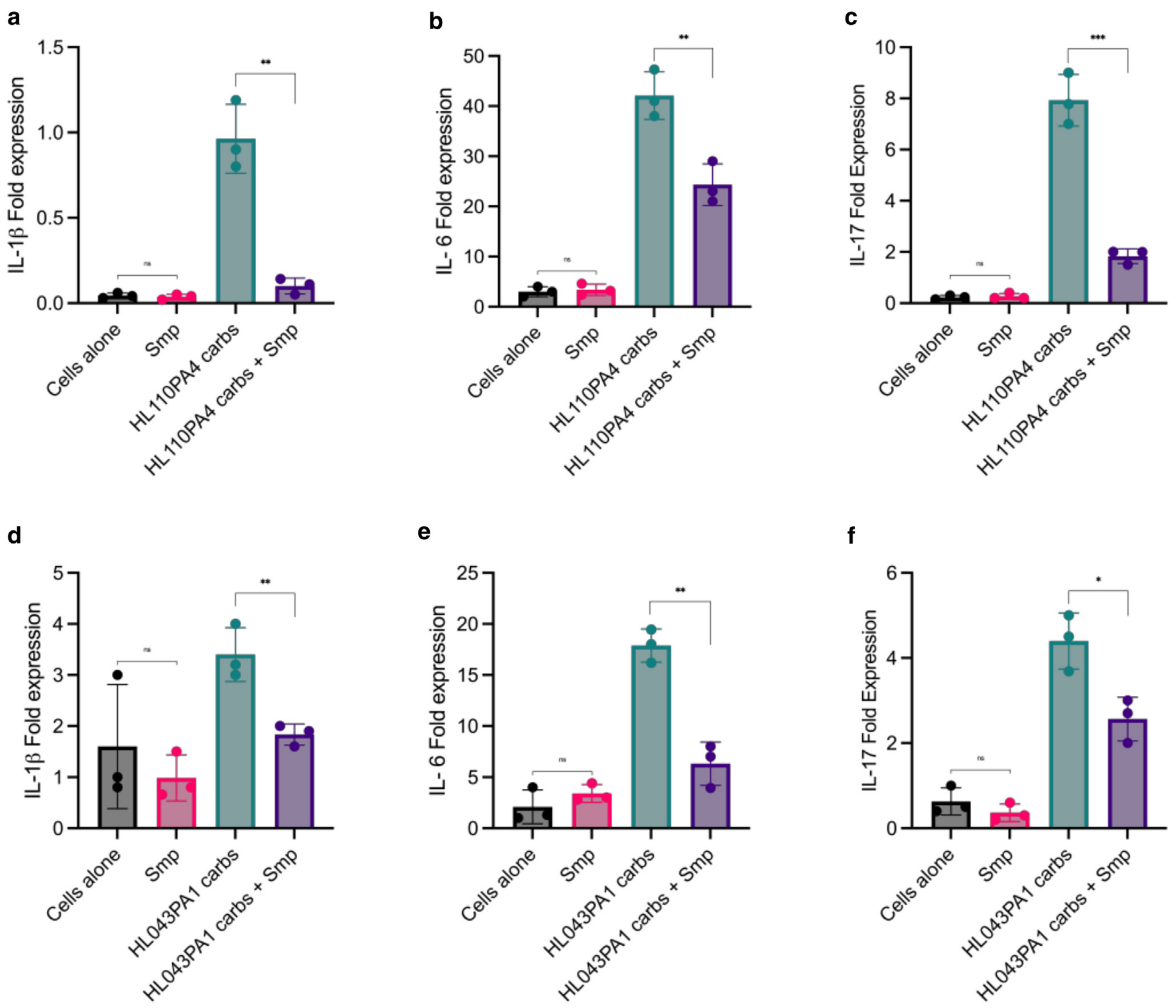

Supplementary Figure S3. *C acnes* carbohydrates have stimulatory potential. (**a**–**c**) PBMCs were stimulated with carbohydrates alone or Smp-treated carbohydrates isolated from *C acnes* strains HL043PA1 and (**d**–**f**) HL110PA4. Real-time PCR of *IL1β*, *IL6*, and *IL17* mRNA expression was analyzed 24 hours after stimulation. Gene expression was normalized to the housekeeping genes *GAPDH* and quantified by the comparative method 2−ΔΔCT. Student's *t*-tests were used to perform the statistical analysis. *$P < .05$, **$P < .01$, and ***$P < .001$. ns, nonsignificant; Smp, sodium meta-periodate.

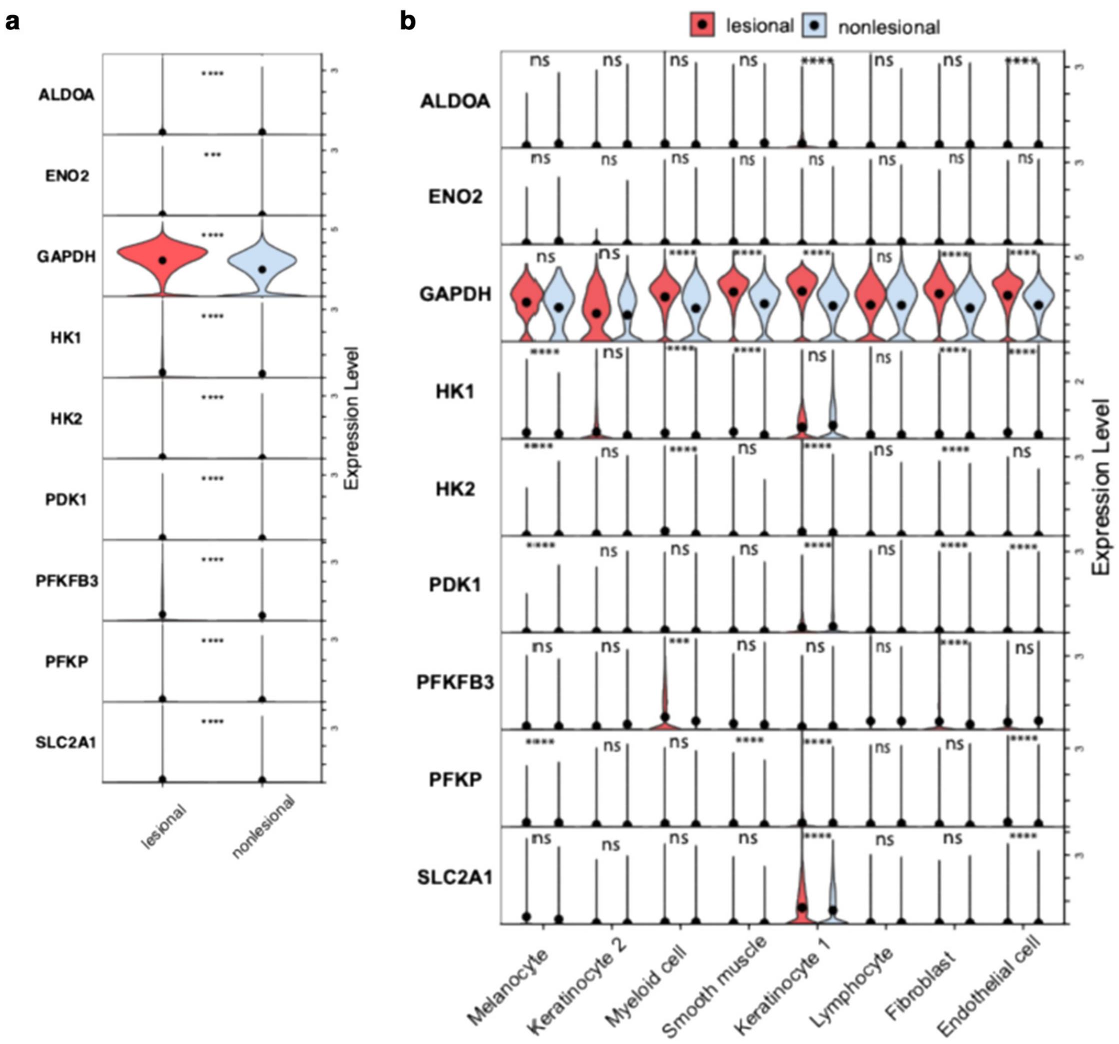

Supplementary Figure S4. Acne triggers enhanced glycolytic activity in lesional skin compared with that in nonlesional skin. (**a**) Dot plots of scRNA-seq data showing the differential gene expression of glycolytic genes in lesional skin compared with those in nonlesional skin. (**b**) scRNA-seq data showing gene expression of glycolytic genes in lesional compared with that in nonlesional samples in melanocytes, keratinocyte 1, keratinocyte 2 (sweat gland cells), myeloid cells, smooth muscle cells, lymphocytes, fibroblasts, and endothelial cells. Wilcoxon tests was used to perform the statistical analysis. ***$P < .001$ and ****$P < .0001$. ns, nonsignificant; scRNA-seq, single-cell RNA sequencing.

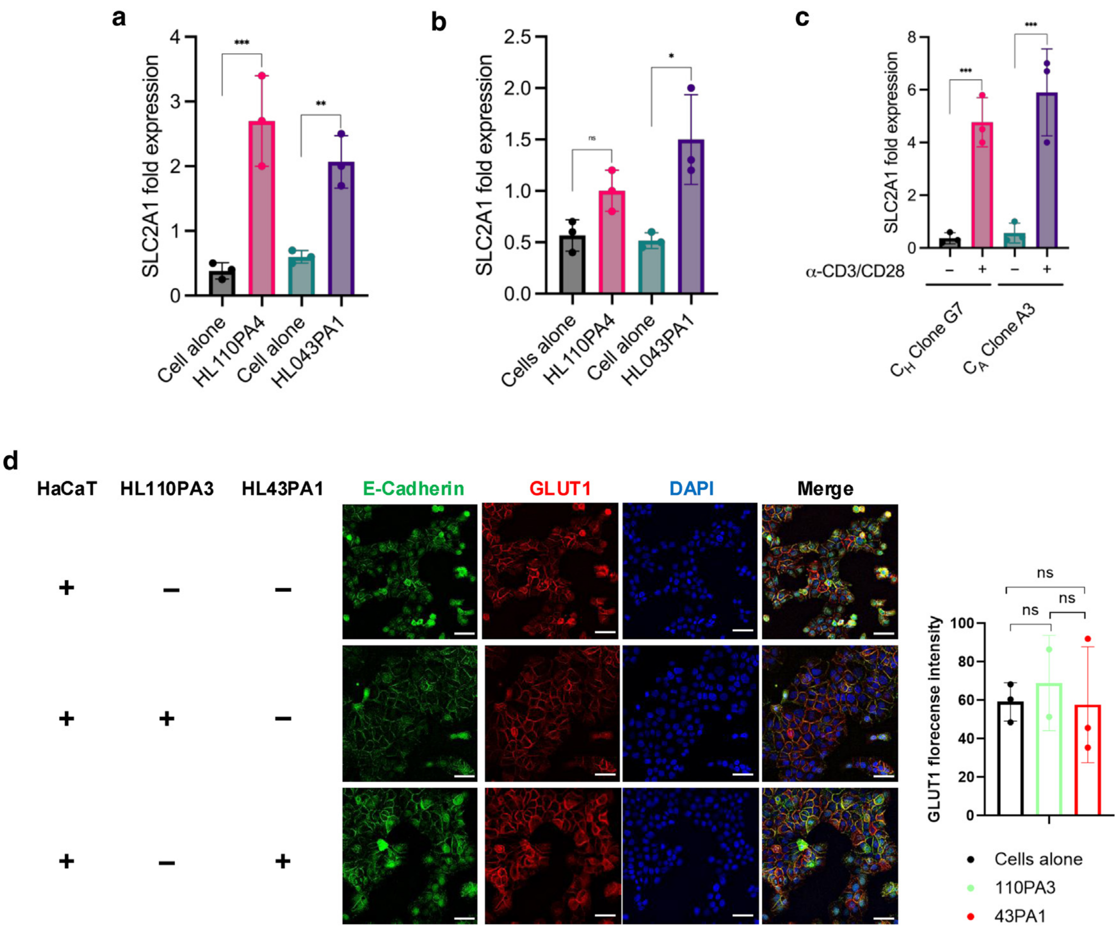

Supplementary Figure S5. Differential expression of SLC2A1 in immune cells. *SLC2A1* mRNA expression was analyzed in different immune cells. (**a**) *SLC2A1* mRNA expression in TREM2 macrophages isolated from human blood; n = 3 donors. (**b**) Expression of *SLC2A1* was also measured in HaCaT cell line. (**c**) Th17 clones were generated as previously described (Agak et al, 2018), and *SLC2A1* mRNA expression was quantified in HL110PA4-specific Th17 clone G7 and HL043PA1-specific Th17 clone A3 after 5-hour stimulation with a-CD3/CD28 antibodies. n = 3. Gene expression was normalized to *GAPDH* and quantified using the 2−ΔΔCT method. Statistical analysis was performed using Student's *t*-test, with significance levels indicated $*P < .05$ and $***P < .001$. (**d**) Fluorescence images of *SLC2A1* (GLUT1) (red) and E-cadherin (green) in HaCaT cells with or without stimulation with *C acnes* HL110PA3 and HL043PA1. Nuclei were stained with DAPI (blue), and E-cadherin was localized at the cell membrane. Bar = 50 μm. The percentage of GLUT1-positive cells within the E-cadherin−positive cell population was quantified using Aivia image analysis and is presented as mean ± SD (n = 3). Statistical analysis indicates no significant differences between conditions. Th17, T helper 17.

Supplementary Table S1. *C acnes* Strains Used in the Study

This Study	Strain	Ribotype: 16S Sequence	Phylotype: SNPs in Core Genome	Multilocus Sequence Typing
Acne-associated strains (C_A)	HL005PA1 HL043PA1 HL096PA1	4.5<br>Acne	IA-2	IA_1 (CC3)<br>Acne
Healthy skin—associated strains (C_H)	HL059PA1 HL110PA3 HL110PA4	6<br>Healthy skin	II, IB-2	II (ST7, CC2)

Supplementary Table S2. Summary of Linkage Types and Biological Implications for C_H and C_A Strains

C acnes Strains	Linkage Type	Possible Biological Implication
HL059PA1	1→4 (linear), 1→6 (branching), terminal (T)	Balanced structure with linear and branched polysaccharides; promotes flexibility and stability; enables bacterial adhesion and immune modulation
HL110PA3	1→4 (linear), 1→3 (branching), 1→6 (complex branching), terminal (T)	Linear backbone with additional branching for flexibility; supports biofilm formation and beneficial skin interactions (de la Fuente-Núñez et al, 2013)
HL110PA4	1→4 (linear), 1→6 (branch points), 1→3 (moderate branching), terminal (T)	Stable yet adaptable structure with balanced branching; enhances biofilm formation, host interactions, and commensal existence
HL005PA1	1→4 (linear backbone), 1→6 (branching), terminal (T)	Stable structure with branching for biofilm formation and immune evasion; promotes adherence to skin cells
HL043PA1	1→4 (linear backbone), 1→6 (branching for biofilm), 1→3 (additional branching), terminal (T)	Rigid backbone with branching for biofilm formation and immune response modulation; enhances pathogenicity in acne
HL096PA1	1→4 (linear backbone), 1→6 (complex branching), 1→3 (minor branching), terminal (T)	Predominantly linear backbone with complex branching; supports biofilm development and interaction with host immune components for survival in lesions

Supplementary Table S3. List of Primers Used in the Study

Gene	Forward Primer Sequence	Reverse Primer Sequence
IL1β	GGACAAGCTGAGGAAGATGC	TCGTTATCCCATGTGTCGAA
IL6	GAAAGCAGCAAAGAGGCACT	TTTCACCAGGCAAGTCTCCT
IL17	ACCAATCCCAAAAGGTCCTC	GGGGACAGAGTTCATGTGGT
CASP1	CCACAATGGGCTCTGTTTTT	CATCTGGCTGCTCAAATGAA
CASP5	AAAGAACAACGTGGCTGGAC	TTGCCCAGGTATTCCAACAT
GAPDH	TGCACCACCAACTGCTTAGC	GGCATGGACTGTGGTCATGAG
SLC2A1	TCTCGAAACTGGGCAAGTCC	TCACCCACATACATGGGCAC
